# Ets-1 Confers Cranial Features on Neural Crest Delamination

**DOI:** 10.1371/journal.pone.0001142

**Published:** 2007-11-07

**Authors:** Eric Théveneau, Jean-Loup Duband, Muriel Altabef

**Affiliations:** 1 Université Pierre et Marie Curie - Paris6, UMR 7622, Laboratoire de Biologie du Développement, Paris, France; 2 CNRS, UMR 7622 Laboratoire de Biologie du Développement, Paris, France; Baylor College of Medicine, United States of America

## Abstract

Neural crest cells (NCC) have the particularity to invade the environment where they differentiate after separation from the neuroepithelium. This process, called delamination, is strikingly different between cranial and trunk NCCs. If signalings controlling slow trunk delamination start being deciphered, mechanisms leading to massive and rapid cranial outflow are poorly documented. Here, we show that the chick cranial NCCs delamination is the result of two events: a substantial cell mobilization and an epithelium to mesenchyme transition (EMT). We demonstrate that *ets-1*, a transcription factor specifically expressed in cranial NCCs, is responsible for the former event by recruiting massively cranial premigratory NCCs independently of the S-phase of the cell cycle and by leading the gathered cells to straddle the basal lamina. However, it does not promote the EMT process alone but can cooperate with *snail-2* (previously called *slug*) to this event. Altogether, these data lead us to propose that *ets-1* plays a pivotal role in conferring specific cephalic characteristics on NCC delamination.

## Introduction

Neural crest cells (NCC) are a transient population of versatile cell types derived from the dorsal neural folds of the developing vertebrate embryo. Their ontogeny is a complex morphogenetic process which encompasses the delicate step of separation from the tight pseudostratified neuroepithelium. This crucial event, called delamination, is characteristic of the NCC population as other components of the early nervous system differentiate and remain confined within the neuroepithelium.

Induction of NCCs appears to take place as early as gastrulation, creating eventually a territory of presumptive NCCs in the dorsal neural tube, at the border between neural plate and epidermis [1]. The most dorsally located of these cells become premigratory NCCs and undergo delamination. This latter process results from an epithelium to mesenchyme transition (EMT) [2], [3], characterized by loss of cell-cell contacts, loss of polarity and acquisition of migratory capabilities [4]. After EMT, NCCs migrate into the periphery, where they differentiate in multiple cell types, prominently neurons and glia of the peripheral nervous system, as well as pigment-producing melanocytes of the skin [3], [5], [6]. In addition, cranial NCCs possess the capability to differentiate into cartilage, bone, connective tissue and smooth muscle, hence constituting the main source of craniofacial structures.

Cranial and trunk NCC delamination events are intrinsically different. Trunk neural tube is flanked by somitic mesoderm and cells emigrate individually in a dripping fashion over a long period of time, more than two days at any given axial level [3], [7]. Recently, mechanisms of action that initiate trunk delamination have been partly unraveled. In the dorsal neural tube, the balance between BMP and its inhibitor Noggin triggers trunk delamination under control of signals coming from somites hence coordinating NCC emigration to somites segmentation [8], [9]. BMP signaling pathway controls G1/S transition in trunk NCCs which delaminate mainly in S-phase [10]. This regulation is pivotal as blockage of G1/S transition prevents delamination from neural tube [11]. In contrast, in head, the neural tube is surrounded by loose mesoderm devoid of somites. Cells pour out as dense, multilayered bulges in a short time scale (approximately 10–15h). So far little is known about signalings that regulate cranial NCC delamination but mechanisms described for trunk are unlikely to apply as inhibition of BMP activity did not prevent cranial delamination [7].

In order to gain insights onto the regulation of cephalic emigration, we sought for genes with cranial-specific expression pattern. Among these, *ets-1*, the founding member of Ets family of transcription factors, is expressed by cranial NCCs just before the onset of emigration and is restricted to cells leaving the neural tube [12]. Furthermore, several lines of evidence implicate *ets-1* in acquisition of cell mobility and invasiveness. During embryonic development, *ets-1* is expressed in tissues exposed to cell movements and scattering such as sclerotome, dermatome and endothelial cells [13]–[18]. Moreover, it endows cells with the capacity to migrate through basement membranes and to invade interstitial space during embryonic angiogenesis and wound healing angiogenesis [19]–[21]. It regulates expression of numerous extra cellular matrix (ECM) components, ECM degrading enzymes and adhesion molecules ([22]–[24] and references therein). *Ets-1* and members of Ets family have also been linked to leukemia, tumor progression and metastasis (for review, [24], [25]). In addition, ETS-1 is also known to regulate genes involved in cell cycle progression such as *p16^ INK4a^*, *p21^WAF1/Cip1^* and *cyclin-d1* [25]–[29]. In light of these properties and of its specific expression pattern, we thus aimed to investigate the role of *ets-1* in NCC development, in particular in cranial versus trunk NCC emigration.

Overall, our studies indicate that in the chick embryo, gathered cranial NCCs delaminate as a multilayered cell population without subjection to G1/S transition of the cell cycle. We demonstrate that the activity of the proto-oncogene *ets-1*, is required for their delamination. Moreover, its ectopic expression allows all neuroepithelial cells (including trunk NCCs) to massively pour out of the neural tube independently of being in S-phase hence mimicking cranial delamination. We show that *ets-1* promotes massive cell recruitment and induces local degradations of the basal lamina, two separable events which are sufficient to initiate ectopic delaminations. In contrast, *ets-1* alone does not provoke epithelium-to-mesenchyme transition (EMT) but can cooperate with *snail-2* (previously called *slug*) to this process. We thus conclude that e*ts-1* confers on NCCs their cranial specific kinetics of delamination.

## Results

### Cranial Neural Crest Cells Delamination is not S-phase Dependent

**Figure 1:**
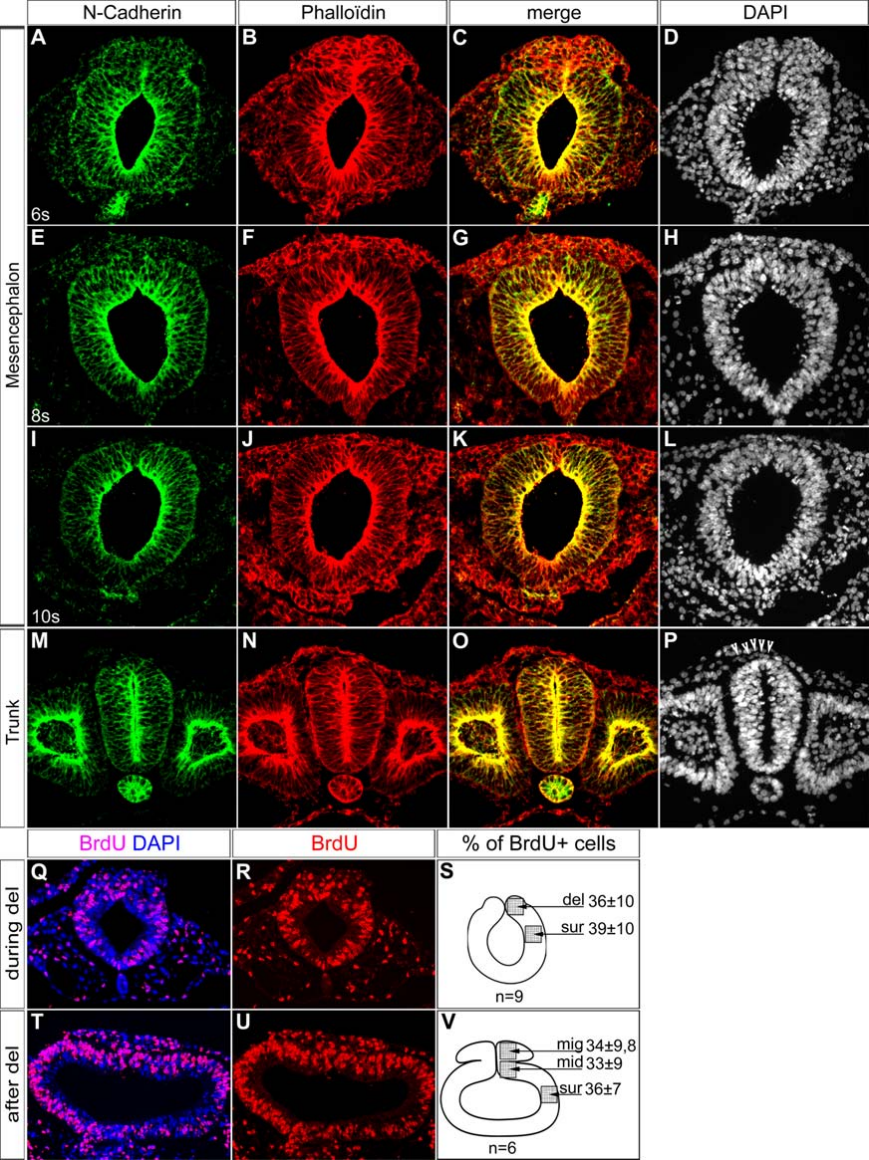
Description of the Cranial Neural Crest Cells Delamination. (A–P) Transversal cryosections (10 µm) of normal chick embryos at stages 6s (A–D), 8s (E–H), 10s (I–L) and HH14 (M–P) at cranial (A–L) and trunk (M–P) levels. Sections were assayed for N-Cadherin expression by immunofluorescence (A, C, E, G, I, K, M, O). The actin microfilaments and the nuclei were stained by Phalloidin (B, C, F, G, J, K, N, O) and DAPI incorporation (D, H, L, P) respectively. During delamination of cranial NCCs there is a massive accumulation of cells in the dorsal part of the neural tube (A–D). In this cell population, colocalisation of N-Cadherin and Phalloidin is lost indicating that they undergo an EMT (C, G, K). By contrast, during trunk delamination, NCCs emigrate one by one. No particular distortion of the dorsal neural tube is detectable (M-P, arrow heads). (Q–V) Analysis of BrdU incorporation in cranial neural tube during and after NCC delamination. Transversal cryosections (5 µm) of stages HH8–9 embryos, during and after delamination, labeled by immunofluorescence using anti-BrdU antibody (Q, R, T, U). Nuclei are stained by DAPI. Percentages of BrdU positive cells in the different zones of the neural tube are represented in diagrams (S,V). Cranial NCCs are not synchronized in S-phase during delamination (Q–R) or migration (T–U). del, delaminating cells; sur, surrounding region; mid, midline region; mig; migrating cells.

In order to better document cranial NCCs delamination, we first analysed the neural tube organization at mesencephalon level using normal chick embryos between stage HH8+ and 10. During the first steps of the delamination process, cranial NCCs are massively gathered together forming a bulge in the most dorsal part of the neural tube (Figures 1A–1L). Noticeably, they continue to express N-Cadherin like neuroepithelial cells, but, its distribution does not coincide with phalloidin staining anymore (Figures 1C, 1G, 1K). This indicates that cell-cell junctions are lost and that cranial NCCs start to undergo EMT. As migration progresses, N-Cadherin expression is gradually lost (Figures 1E, 1I). By contrast, trunk NCCs leave the neural tube progressively, one after another, they loose N-Cadherin expression at the onset of delamination (Figures 1M-1P, [30]) and leave the neural tube only when they are in S-phase [11]. We thus examined BrdU incorporation in cranial NCCs during their delamination. Control embryos were exposed between stages HH8 and 9 to BrdU for one hour. BrdU positive cells were counted in different parts of the neural tube corresponding to delaminating cranial NCCs (del), early migrating cranial NCCs (mig), dorsal midline of the neural tube after delamination (mid), and regions surrounding neural crest territory (sur). In all these areas, we never detected any synchronization in S-phase (Figures 1Q–1V) showing that delaminating cranial NCCs do not leave the neural tube preferentially in S-phase. Moreover, there is no depletion of BrdU positive cells in the dorsal neural tube after delamination (Figures 1T–1V) contrary to trunk levels. Cranial NCCs delamination is therefore characterised by the exit of clustered cells from dorsal neural tube which are not synchronized in S-phase.

### Ets-1 Activity During Delamination is Required at Cranial Levels Only

**Figure 2:**
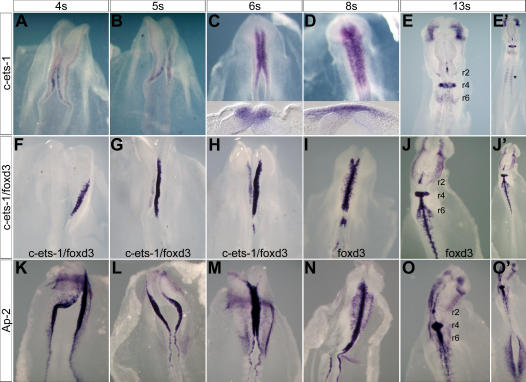
*Ets-1* Expression Occurs Later and in a More Restricted Area than Those of Foxd3 and AP2. (A–O') Whole in situ hybridization of normal chick embryos at stages 4s–13s using *c-ets-1* (A–H), *foxd-3* (F–J') and *ap-2* (K–O') probes. (F–H) Embryos were cut along the rostro-caudal axis. The left and right sides of the neural tube were treated independently with *c-ets-1* and *foxd-3* probes. *C-ets-1* expression (A–B, F–G) begins after *foxd-3* (F–G) and *ap-2* (K–L) expressions. It is restricted to cells leaving the neural tube (C–D, insets) and cranial region (compare E–E' to J–J' and O–O'). * indicates *ets-1* expression in the sclerotome. r, rhombomere.

**Figure 3:**
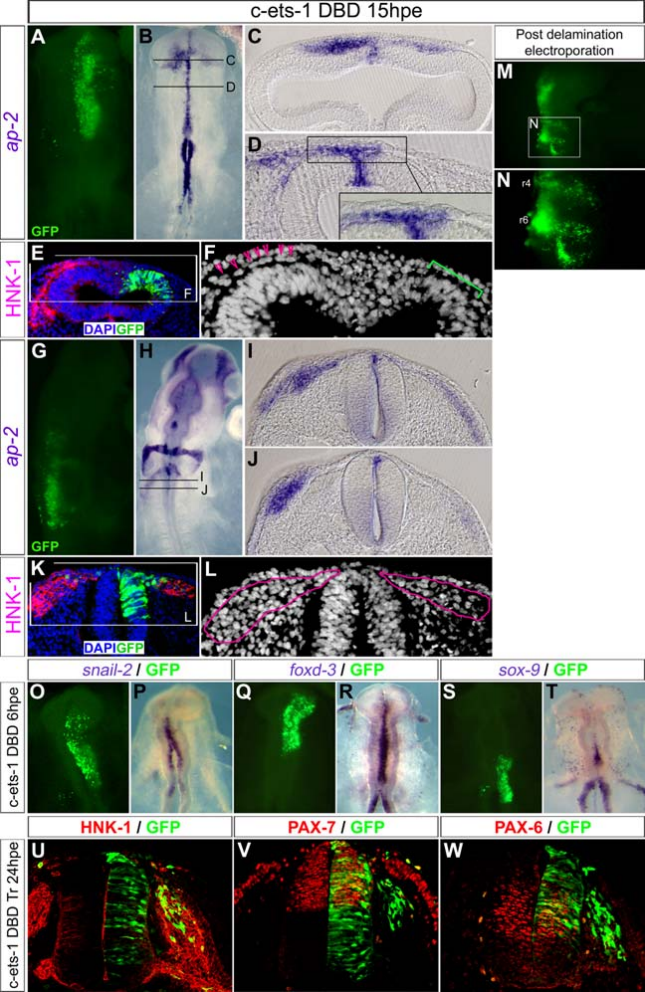
Ets-1 Activity Is Required for Cranial but not Trunk Delamination. (A–L) Analysis of cranial NCCs delamination in HH7-9 chick embryos electroporated by *c-ets-1 DBD* and harvested at 15 hours post electroporation (hpe). (A–D; G–J) Whole mount in situ hybridization using *ap-2* probe. GFP expression in (A) and (G) indicates the electroporation zone. (C–D) and (I–J) are vibratome sections (30 µm) of embryos presented in (B) and (H) respectively. (E–F; K–L) Transversal cryosections (14 µm) labeled by immunofluorescence using anti-HNK-1 antibody and nuclear-stained by DAPI incorporation. Electroporated cells are detected by GFP expression. Pink broken lines in (L) outline the NCCs. (M–N) Analysis of NCC migration in embryos electroporated in the rhombencephalon at stage HH10+ when the delamination is in progress and harvested at 15hpe (stage HH14). (O–T) Analysis of NCC specification in chick embryos electroporated by *c-ets-1 DBD* at stage HH7-9 and harvested at 6hpe. GFP expression in (O), (Q) and (S) indicates the electroporation zone of the embryos presented in whole mount in situ hybridization using *snail-2*, *foxd-3* and *sox-9* probes in (P), (R) and (T) respectively. (U–W) Analysis of trunk NCCs delamination in chick embryos electroporated by *c-ets-1 DBD* harvested at 24hpe. Transversal cryosections (10 µm) were labeled by immunofluorescence using anti-HNK-1 (U), anti-Pax-7 (V) and anti-Pax-6 (W) antibodies. Expression of *c-ets-1 DBD* in the head leads to a decrease or a lack of *ap-2* (B–D, H–J) and HNK-1 expression (E, K). At cellular level, there is either a reduction of the size of the NCC stream on the electroporated side (L) or even a lack of NCCs between the ectoderm and the neural tube (E; F, green staple) while on control side NCCs are normally localized (B–D, ap-2 staining; F, pink arrow heads; L, left pink lasso). Data shown in A–D, E–F, G–J, K–L come from four distinct embryos respectively. This effect is restricted to the delamination step since inhibition of ETS-1 activity after the delamination does not affect the migration (M–N). Finally, *c-ets-1 DBD* does not prevent NCC specification as *snail-2* (O–P), *foxd-3* (Q–R) and *sox-9* (S–T) remain expressed in the neural folds on both electroporated and control sides. At trunk level, misexpression of *c-ets-1 DBD* has no effect on dorso-ventral patterning, or on NCCs delamination and migration (U–W). r, rhombomere.

We sought for gene candidates which expression could sustain such a specific delamination process. In this context, *ets-1* is of particular interest since it is expressed in NCCs specifically at cranial level ([12] and Figures 2A–2E') with an extremely dynamic pattern of expression which matches the delamination process (Figures 2A–2D). *Ets-1* transcripts can first be detected at 4s-stage in the cranial neural folds of chick embryo. From 5s-stage, its transcription rapidly increases as delamination takes place and is restricted to delaminating cells, being absent in the remaining neuroepithelial cells (see insets in Figures 2C–2D). Furthermore, *ets-1* expression begins after the onset of expression of early NCCs markers such as *foxd-3* (Figures 2F–2H) and *ap-2* (Figures 2K–2M). In addition, *ets-1* expression is restricted to the cranial region (Figures 2E–2E') whereas *foxd-3* (Figures 2J–2J') and *ap-2* (Figures 2O–2O') are both expressed in head and trunk NCC. These data argue in favour of a specific role of *ets-1* into the cranial NCCs delamination process. In order to lend support to this hypothesis, we analysed the effects of a transdominant negative form of *ets-1* consisting only in the DNA binding domain (*c-ets-1* DBD) both at cranial and trunk levels. The resulting protein competes with endogenous chick-ETS-1 for binding the DNA but, lacking its transactivation domains, it does not regulate genes expression. We first misexpressed *c-ets-1* DBD in the cranial regions of stages HH7-9 embryos (Figures 3A–3L, n = 8). At 15 hours after electroporation (hpe), in regions which normally express *ets-1* ([12] and personal data), there is a dramatic reduction of HNK1 and *ap-2* positive cells invading the migration pathways on the electroporated mesencephalon (Figures 3A–3F, n = 4) or rhombencephalon (Figures 3G–3L, n = 4). It suggests a great decrease or the absence of delaminating NCCs. However, *ap-2* expression in the dorsal neural tube is not affected suggesting that NCCs specification is not inhibited (Figures 3C–3D, 3I–3J). To further support that *c-ets-1* DBD does not affect the initial NCCs specification, we monitored *snail-2* (Figures 3O–3P, n = 4), *foxd-3* (Figures 3Q–3R, n = 6) and *sox-9* (Figures 3S–3T, n = 6) expression patterns during this process at 6hpe. None of these genes is downregulated after *c-ets-1* DBD electroporation indicating that ETS-1 activity is not required for NCC specification. To also rule out the possibility that ets-1 could be involved in NCC migration, we misexpressed *c-ets-1* DBD, at later stages, in the rhombencephalon of stage HH10+ embryos and analyse the NCC migration at 15hpe. No inhibition is observed from either rhombomeres r4 or r6 NCC streams (Figures 3M–3N) showing that loss of ETS-1 activity does not antagonize NCC migration. Finally, in order to exclude non-specific roles of the c-ets-1 DBD, we also electroporated the construct in the trunk dorsal neural tube. At 24hpe, the dorso-ventral patterning is not disturbed (Figures 3U–3W; Pax6, n = 4, Pax7, n = 4), and, the NCCs delaminate and migrate away normally as a population of dissociated cells expressing HNK1 (Figure 3U; n = 5). Altogether, these data show a specific requirement of *ets-1* in cranial NCCs delamination.

### *Ets-1* is Sufficient to Provoke a Cranial-Like NCCs Delamination in Dorsal Trunk Neural Tube

**Figure 4:**
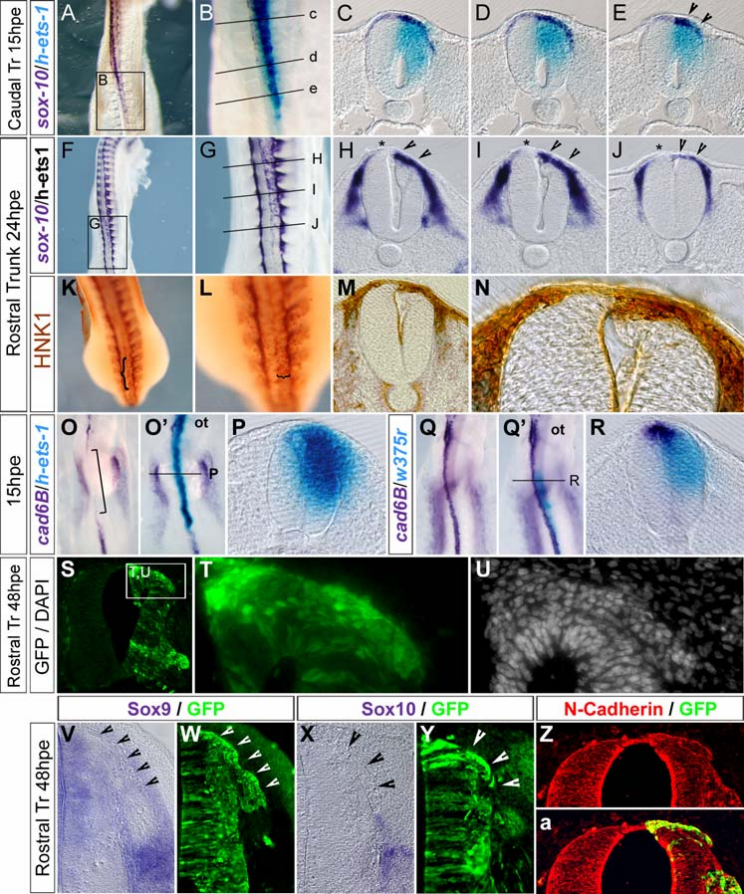
Trunk NCC Delamination Occurs Prematurely is Amplified and Prolonged by *h-ets-1* Misexpression. (A–a) Analysis of the effects of *h-ets-1* misexpression in trunk dorsal neural tube assayed at 15hpe (A–E, O–R), 24hpe (F–N) and 48hpe (S–a). (A–J, O–R) Whole mount in situ hybridization using *sox-10* (A–J, dark blue), *cadherin-6B* (O–R, dark blue) and *h-ets-1* (A–E, O–R, light blue) probes. (K–N) Wholemount immunostaining using anti HNK1 antibody. (C–E, H–J, M–N, P, R) Vibratome sections (30 µm) of embryos presented in (B), (G), (L), (O') and (Q') respectively. (V–Y) In situ hybridization on transversal cryosections (20 µm) using *sox-9* (V–W) and *sox-10* (X–Y) probes. (Z–a) Transversal cryosections (10 µm) immunolabeled using anti-N-Cadherin antibody. Electroporated cells are detected by GFP expression (S–T, W, Y, a) or DAPI staining (U). At 15hpe in *h-ets-1* caudally transfected embryos, *sox-10* trunk NCCs delaminate precociously (A–B, E, arrow heads). Besides, more rostrally, the outflow is increased (C–D) compared to contralateral side and is associated with a loss of *cadherin-6B* expression (O–P). At 24hpe, at level where delamination is already completed on the control side (H–J, asterisks), *h-ets-1* expression prolongs delamination of a massive amount of *sox-10* (H–J, arrow heads) and HNK-1 (M–N) positive NCCs. At 48hpe, *h-ets-1* transfected cells are still able to leave the dorsal neural tube as a multilayered wave (S–U) but they fail to express NCCs markers such as *sox-9* (V–W), *sox-10* (X–Y) and keep a strong expression of N-Cadherin (Z–a). ot, otic vesicle.

In order to test whether *ets-1* expression plays an important role in conferring specific cranial NCCs delamination features, we misexpressed the human form of *ets-1* (*h-ets-1*) in dorsal trunk neural tube in stage HH10 embryos. At 15hpe, we observed more *sox-10* expressing NCCs emigrating from the electroporated side compared to the control side (Figures 4A–4D; n = 7). Moreover, at more caudal level, where the endogenous delamination has not started yet, we observed a premature exit of s*ox-10* positive NCCs in the electroporated side, in contrast with the control side where *sox-10* expression is barely detectable (Figures 4A, 4E; n = 7). Electroporation of an inactive form of *ets-1* (*w375r*) in the same conditions has no effect (data not shown). This shows that *ets-1* expression can prime NCCs delamination and in addition increase the flow of delaminating cells. This effect is associated with a strong decrease of *cadherin-6B* expression (Figures 4O–4P; n = 5), which is normally lost by the NCCs leaving the neural tube [31]. Expression of *w375r* does not affect it (Figures 4Q–4R). Therefore, this suggests that *ets-1* forces the dorsal premigratory NCCs to massively leave the neural tube. At 24hpe, the trunk NCC emigration is enlarged as evidenced by a thick flow of *sox-10* (Figures 4F–4J, arrow heads; n = 4) and HNK1 (Figures 4K–4N; n = 2) positive cells delaminating from the dorsal neural tube. At rostral trunk level, NCCs delamination persists whereas the endogenous delamination is already completed on the control side (Figures 4H–J, asterisks; n = 4). However, at 48hpe, cells which leave the neural tube under *ets-1* expression do not display NCCs characteristics anymore. Delaminating electroporated cells are organized as a tongue at the top of the dorsal neural tube (Figures 4S–4U; n = 7). They express high level of N-Cadherin (Figures 4Z–4a) whereas its specific repression is required for trunk NCCs departure [30] and fail to express NCCs markers such as *sox-9* (Figures 4V–4W), s*ox-10* (Figures 4X–4Y) or HNK1 (Figures 6S–6T). Altogether these results indicate that *ets-1* expression leads to precocious, enhanced and prolonged delamination of trunk NCCs which delaminate gathered as a multilayer stripe of cells instead of one by one progressively.

**Figure 5:**
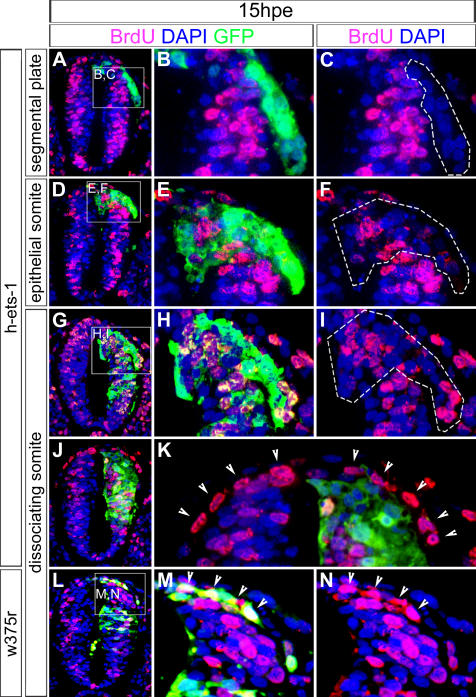
*Ets-1* Misexpression Emancipates Trunk NCC Delamination from Subordination to Successful G1/S Transition. (A–N) Analysis of the effects of *h-ets-1* misexpression in trunk dorsal neural tube on cell cycle assayed at 15hpe. Immunofluorescence labeling using anti-BrdU antibody of transversal cryosections (5 µm). Nuclei are stained by DAPI incorporation. Dotted lines in (C), (F), (I) indicate delaminating transfected area as defined by GFP expression (B, E, H). Trunk NCCs emigrating precociously from dorsal electroporated neural tube opposite segmental plate (A–C) or the first epithelial somite (D–F) are not synchronized in S-phase. Similarly, opposite dissociating somites (G–I), *h-ets-1* misexpression in the dorsal neural tube leads to increased NCC delamination of a mix of BrdU positive and negative cells. In contrast, trunk NCCs are predominantly in S-phase when *h-ets-1* misexpression does not target the most dorsal territory (J–K, arrow heads) or when NCCs are transfected by *w375r* (L–N, arrow heads).

It thus raised the question of whether these cells are still subordinate to successful G1/S transition to delaminate as in normal conditions. We exposed embryos electroporated with *h-ets-1* to BrdU for one hour at 15hpe. At all considered levels, BrdU positive emigrating cells are mingled with high proportion of negative cells indicating a lack of synchronization at the time of departure (Figures 5A–5I; segmental plate, n = 4; epithelial somite, n = 2; dissociating somite, n = 4). By contrast, untransfected NCCs are predominantly in S-phase (Figures 5J–5K), consistent with previous data [11]. Likewise, misexpression of *w375r* in trunk NCCs does not abolish the synchronization in S-phase (Figures 5L–5N). Therefore, when expressing *ets-1*, trunk NCCs are not bound to G1/S transition in order to delaminate, consequently the delamination rate is enhanced.

Overall, our data show that *ets-1* is sufficient to convert the slow dripping delamination shaped by subjection to the cell cycle and characteristic of trunk NCCs into a massive cranial-like emigration emancipated from links to G1/S transition.

### *Ets-1* Promotes Ectopic Cells Emigrations from the Neuroepithelium without Inducing Neural Crest Fate

To better appreciate the capabilities that *ets-1* could confer on the neuroepithelial cells autonomously, we decided to analyse the effects of its ectopic expression within the intermediate to ventral parts of the neural tube, regions which cannot produce NCCs in normal conditions.

**Figure 6:**
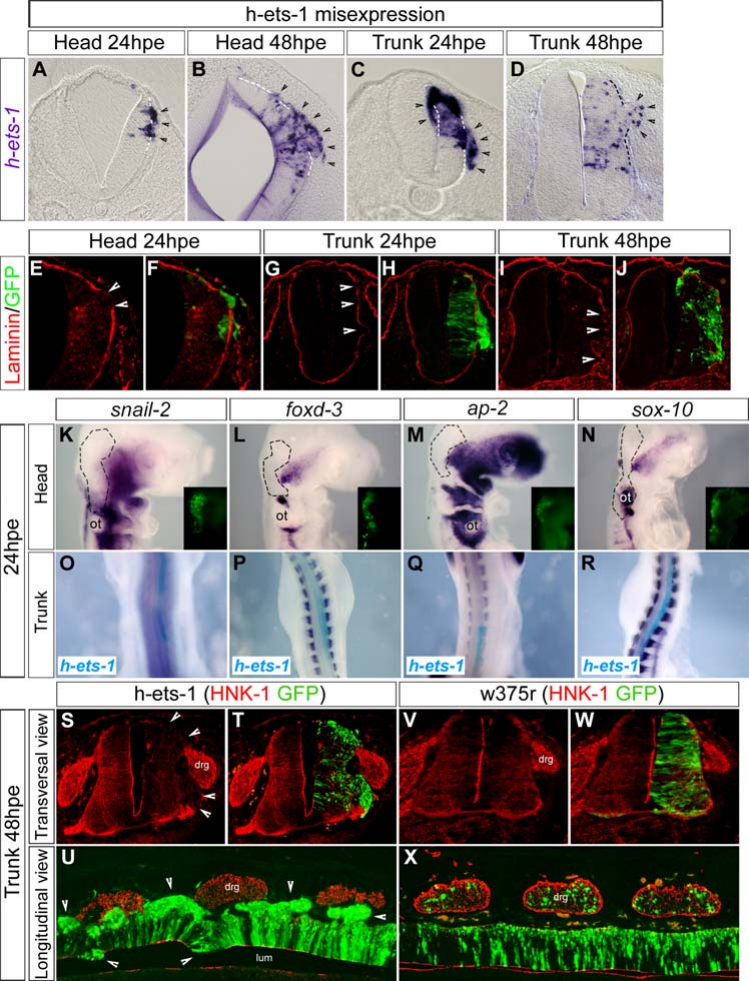
*Ets-1* Misexpression Triggers Ectopic Delamination without Inducing Neural Crest Fate. (A–J) Analysis of the effects of *h-ets-1* misexpression in intermediate to ventral neural tube at 24hpe (A, C, E–H) and 48hpe (B, D, I–J); at head (A–B, E–F) and trunk (C–D, G–J) levels. (A–D) Vibratome sections (30 µm) of whole mount in situ hybridization using *h-ets-1* probe. At 24hpe, misexpression of *h-ets-1* leads to ectopic delaminations towards basal or luminal sides (A, C, arrow heads; dotted lines indicate the neural tube limit). At 48hpe, the phenomena is stronger, involving more cells leaving the neural tube in both head (B) and trunk (D) as compact bulges of cells. (E–J) Transversal cryosections (10 µm) labeled with anti-Laminin antibody. Electroporated cells degrade the basal lamina (arrow heads) before invading the ECM. (K–X) Analysis of NCC fate in ectopic delaminating cells. (K–R) Whole mount in situ hybridization with *snail-2* (K–O, dark blue), *foxd-3* (L–P, dark blue), *ap-2* (M–Q, dark blue), *sox-10* (N–R, dark blue) and *h-ets-1* (O, P, Q, R, light blue) probes. Dotted lines in (K), (L), (M), (N) indicate the transfected area as defined by GFP expression (insets in K, L, M, N). (S–X) Immunofluorescence labeling with anti-HNK-1 antibody on transversal (S–T, V–W) and longitudinal (U, X) cryosections (10 µm). At 24hpe, misexpression of *h-ets-1* in head or trunk does not ectopically activate *snail-2*, *foxd-3*, *ap-2* or *sox-10* (K–R). Furthermore, at 48hpe ectopic cells (including cells emerging from the dorsal part of the neural tube) never express HNK-1 (S–U, arrow heads). Misexpression of *w375r* has no effect (V–X). drg, dorsal root ganglia; lum, lumen; ot, otic vesicle.

As early as 12hpe, both at cranial and trunk levels, *h-ets-1* misexpression leads to small ectopic clusters of cells emerging from the neural tube (n = 13, not shown). At 24hpe and 48hpe, the phenotype is amplified and compact heaps of clustered cells are detected bulging out through the extracellular matrix or towards the lumen (Figures 6A–6D, 24hpe, n = 26, 48hpe, n = 18). Misexpression of *w375r* has no effect indicating that the observed phenotype is due to specific transcriptional activation of *ets-1* targets (n = 21, not shown). At 24hpe and 48hpe, these ectopic bulges of cells are associated with local degradations of the basal lamina (Figures 6E–J, arrow heads, n = 8) hence confirming the initiation of a delamination process. Given that delamination is a trademark of NCCs compared with others neuroepithelial cells, we assessed whether ectopic delaminating cells induced by *h-ets-1* at intermediate to ventral level of the neural tube did also express NCCs markers. At 24hpe, at both cranial and trunk levels, *h-ets-1* does not induce expression of *snail-2* (Figures 6K, 6O, n = 8), *foxd-3* (Figures 6L, 6P, n = 8), *ap-2* (Figures 6M, 6Q, n = 8) or *sox-10* (Figures 6N, 6R, n = 10). Similarly, we never detected HNK-1 immunoreactivity in transfected neural tubes or in intermediate delaminating electroporated cells at 24 or 48hpe (Figures 6S–6X, n = 7). To turn down the possibility of transient induction of NCC markers, we analysed *snail-2* and *sox-10* expressions at earlier time points and did not find any upregulations (data not shown).

Therefore, *ets-1* expression within the neural tube initiates ectopic delamination independent of NCCs fate.

### *Ets-1* Induces Cell Mobilization but not Cell Dispersion

**Figure 7:**
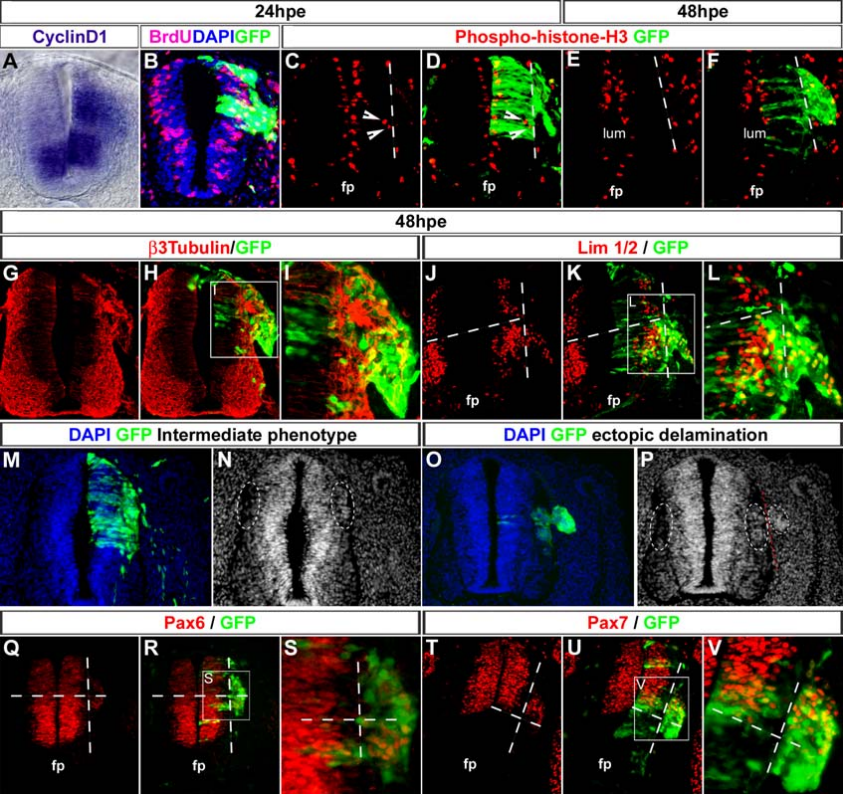
*Ets-1* Misexpression Leads to Massive Cell Movements within the Neuroepithelium. (A–V) Analysis of the effects of *h-ets-1* misexpression in intermediate to ventral neural tube at 24hpe (A–D) and 48hpe (E–V). (A) Vibratome section (30 µm) of whole mount in situ hybridization using *cyclin-d1* probe. (B–L, Q–V) Immunofluorescence on cryosections (10 µm) with anti-BrdU (B), anti-phosphohistoneH3 (C–F), anti-β3-Tubulin (G-I), anti Lim-1/2 (J–L), anti-Pax-6 (Q–S), anti-Pax-7 (T–V) antibodies. (M–P) Nuclei are stained with DAPI. *H-ets-1* misexpression leads to ectopic activation of *cyclin-d1* expression without affecting equilibrium between cell proliferation (B–F) and cell differentiation (G–L). Ectopic *h-ets-1* expression provokes cell accumulation close to the basal side of the neural tube (M–P). Interestingly, cell recruitment is detectable even when the phenotype is not strong enough to lead to ectopic delamination (M–N). These cell movements of neuroepithelial cells occur along the apico-basal axis of the neural tube and do not disturb dorso-ventral patterning (Q–V). fp, floor plate; lum, lumen.

*Ets-1* is known to be involved in tumorigenesis. Therefore, to better understand the nature of the ectopic clusters and investigate their possible tumor-like nature, we decided to analyse the impact of *h-ets-1* expression on neural cells proliferation. Despite an upregulation of *cyclin-d1* expression, a direct target of *ets-1* [29], on the electroporated side (Figure 7A, n = 3), ectopic delaminating cells are not mainly in S-phase (Figure 7B, n = 3) or in mitosis (24hpe, Figures 7C–7D, n = 2 ; 48hpe, Figures 7E–7F, n = 4). Accordingly, endogenous expression of genes expressed in proliferative (*sox-2*, n = 2, *sox-9*, n = 3; data not shown) or differentiated cells (β3-Tubulin, Figures 7G–7I, n = 3; Lim-1/2, Figures 7J–7L, n = 3) are not affected. Furthermore, proliferative and non-proliferative cells are equally mingled together in ectopic delaminating clusters (Figures 7G–7L). Taken together, these data indicate that *ets-1* expression has no effect on cell proliferation.

Interestingly, even though the proliferation was not increased, we noted ectopic cells in M-phase located at the basal side of the neural tube at 24hpe (Figures 7C–7D, arrow heads). In addition, at 48hpe, we observed accumulations of cell nuclei between the non-proliferating region of the neural tube and the basal lamina, an area normally largely deprived of nuclei (Figures 7M–7P, n = 12). This led us to hypothesize that cells movements within the neural tube might occur under *ets-1* expression. To test out this hypothesis, we used regionalized markers such as Pax-6 (Figures 7Q–7S, n = 6) and Pax-7 (Figures 7T–7V, n = 6). At 48hpe, Pax-6 and Pax-7 positive cells can be detected within the ectopic clusters but only in register with their endogenous region of expression. It hence gives rise in some cases to mixed Pax-6-low/high expressing clusters or mixed Pax-7-expressing/non-expressing clusters (see high magnifications in Figures 7S and 7V). These results show that Pax-6 and -7 are not ectopically induced in ectopic clusters and that regionalization of the neural tube is conserved in them. We thus conclude that *ets-1* induces massive cell movements along apico-basal axis of the neuroepithelium and recruits neuroepithelial cells for subsequent delamination initiated by local disruption of the basal lamina. It is interesting to note that cell mobilization and basal lamina degradation are two separate events since if we use a non-phosphorylable form of *ets-1*, cell mobilization occurs without a systematic degradation of the basal lamina (n = 9, not shown).

**Figure 8:**
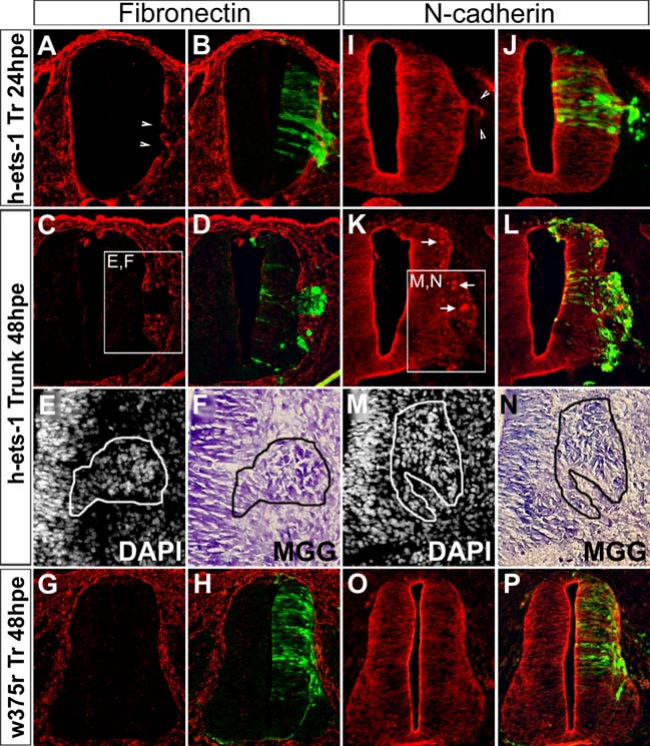
*Ets-1* Misexpression Promotes Delamination without Inducing Epithelium to Mesenchyme Transition. (A–P) Analysis of the effects of *h-ets-1* misexpression in intermediate to ventral neural tube at trunk levels at 24hpe (A–B, I–J) and 48hpe (C–H, K–P). Transversal cryosections (10 µm) labeled by immunofluorescence with anti-Fibronectin (A–D, G–H), anti-N-cadherin (I–L, O–P) antibodies, by DAPI incorporation (E, M) and by histological staining with May-Grünwald Giemsa (MGG) (F, N). *H-ets-1* electroporated cells invade the extracellular matrix without producing Fibronectin (A–D, arrow heads) and remain strongly attached to each others by N-cadherin (I–L, arrow heads) at 24hpe (A–B, I–J) and 48hpe (C–D, K–L). Accumulations of N-Cadherin are observed within the core of the ectopic clusters at 48hpe (K–L, white arrows). Nuclear detection (E, M) and histological staining (F, N) of sections presented in (C) and (K) confirm the high cellular density in the ectopic clusters (E–F, M–N, white and black lines). Cells transfected by *w375r* exhibit normal behavior (G–H, O–P). Tr, trunk.

**Figure 9:**
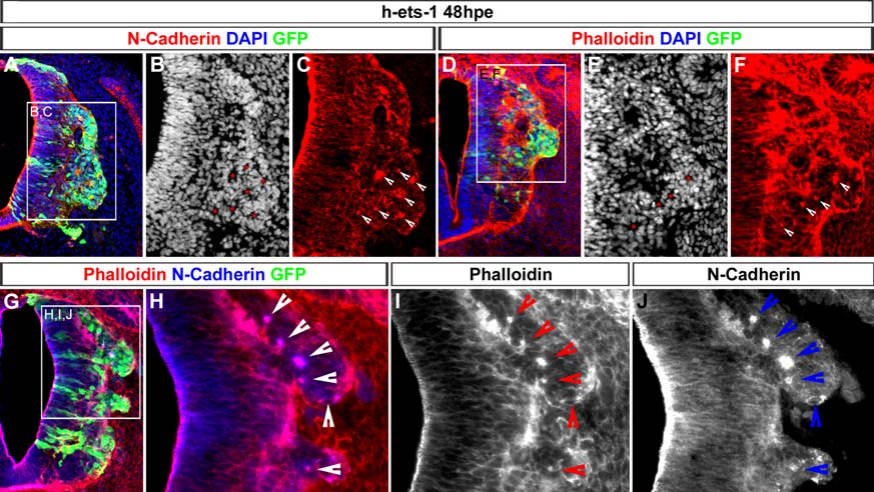
Ectopic Electroporated Cells Are Still Attached by Functional Cell-Cell Junctions. (A–J) Analysis of the effects of *h-ets-1* misexpression in intermediate to ventral neural tube at 48hpe. Transversal cryosections (10 µm) labeled by immunofluorescence with anti-N-Cadherin antibody (A–C, red; G–J, blue). Actin microfilaments and nuclei are stained with Phalloidin (D–I) and DAPI incorporation (A–F) respectively. Electroporated cells detected by GFP expression (A, D, G) are organized around dots of high N-Cadherin expression (A–C, red dots, white arrows) or high Phalloidin staining (D–F, red dots, white arrows). N-Cadherin and Phalloidin perfectly match to each other (G–J, arrow heads) indicating that N-Cadherin expressed by the electroporated cells is involved in functional cell-cell junctions.

Since the ectopic clusters of electroporated cells remain close to the neural tube and no migrating electroporated cells are detected far away, it seems that *ets-1* does not promote acquisition of migratory capabilities. Migrating cells are normally individually surrounded by ECM. In contrast, clusters of delaminating electroporated cells are completely devoid of Fibronectin (head not shown, trunk Figures 8A–8D, n = 7). In addition, these cells retain a strong expression of N-Cadherin (head not shown, trunk Figures 8I–8L, n = 7), similar to the adjacent neuroepithelium, which suggests cohesive relationships between them. Accordingly, the bulges are formed of a high density of cells (Figures 8E–8F, 8M–8N) comparable to the density observed in the neuroepithelium. In order to test out whether the N-Cadherin was involved in functional structures, we analysed the distribution of the actin microfilaments by Phalloidin staining. Within the ectopic bulges, there are some hot spots of N-Cadherin expression (Figure 8K, arrows, n = 7). This particular distribution of N-Cadherin correlates with a specific organization of the electroporated cells around the hot spots (Figures 9A–9C, 9G–9J) which coincides with the distribution of the actin microfilaments (Figures 9D–9F, 9G–9J, n = 8). These results indicate that, in the electroporated cells, N-Cadherin is still involved in functional cell-cell junctions and strongly argue against EMT and migration abilities. This observation is confirmed by the fact that electroporated cells do not upregulate various molecules involved in NCC EMT or expressed by mesenchymal cells such as RhoB ([32]; n = 12, not shown), Cadherin-7 ([31]; n = 7, not shown), activated β1-Integrin ([33]; n = 8, not shown), Tenascin ([34]; n = 4, not shown) and β3-Integrin ([35]; n = 3, not shown). All these data indicate that the ectopic delamination process initiated by *ets-1* occurs without an EMT.

Overall, this suggests that, during the normal delamination of cranial NCCs, *ets-1* massively recruits cells, initiates delamination but is not able to orchestrate EMT which achieves the process.

### *Ets-1* and *Snail-2* Cooperate to Achieve a Full Delamination Process

**Figure 10:**
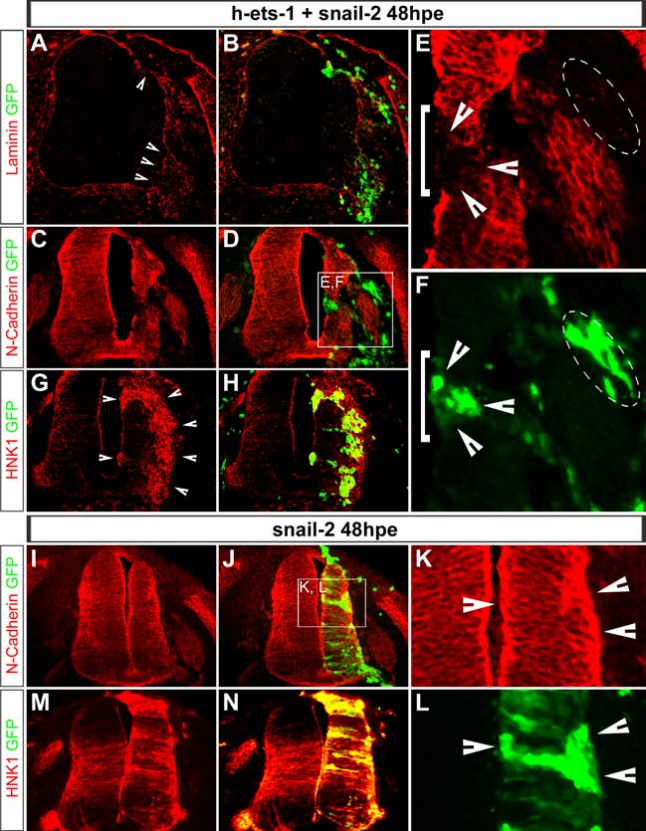
*Ets-1* and *Snail-2* Cooperate to Achieve Delamination. (A–N) Analysis of the effects of *h-ets-1* and *snail-2* coelectroporation (A–H) and *snail-2* alone (I–N) in intermediate to ventral neural tube at 48hpe. Transversal cryosections (10 µm) labeled with anti-Laminin (A–B), anti-N-Cadherin (C–F, I–L) and HNK-1 (G–H, M–N) antibodies. Co-electroporated cells degrade the basal lamina (A–B), lose N-Cadherin expression (C–F, white arrow heads and dotted line), cell-cell junctions at the apical side (white bracket) and strongly express HNK1 (G–H). These cells emigrate from the tube as a population of dissociated cells. *H-ets-1* and *snail-2*, electroporated together, are able to promote EMT and migratory NCCs identity. Conversely, *snail-2* electroporation does not affect either N-Cadherin expression or distribution (I–L, white arrow heads). Electroporated cells are unable to undergo EMT and then remain in the neural tube. However, *snail-2* electroporation leads to massive ectopic activation of HNK-1 (M–N) all along the dorso-ventral axis of the neural tube.

As the cranial NCCs perform an EMT during their normal development, we looked for gene able to achieve ectopic delaminations initiated by *ets-1*. Interestingly, *snail-2* has been described to increase the total amount of emigrating cranial NCCs but, strikingly, does not affect trunk NCCs [36]. Moreover, in contrast to *ets-1*, *snail-2* is unable to induce neuroepithelial cells from intermediate to ventral level of the neural tube to delaminate [36], [37]. These results suggest that *snail-2* could be only active in cells expressing *ets-1*. Therefore, we analysed its ability to cooperate with *ets-1* to promote accomplished delamination of trunk neuroepithelial cells including mesenchymalisation. We hence coelectroporated *h-ets-1* and *snail-2* at trunk level in intermediate to ventral neural tube and analysed the effects at 48hpe. Local degradations of the basal lamina are detected in association with ectopic delaminations as expected from *h-ets-1* ectopic expression (Figures 10A–10B, n = 4). However, in contrast to *h-ets-1* electroporation alone, coelectroporated cells do not express N-Cadherin either within or outside the neural tube (Figures 10C–10F, see arrow heads in E and F, n = 3) and the ectopic delaminating cells invade the ECM as a population of dissociated cells (Figures 10E–10F, n = 4). Furthermore, electroporated cells strongly express HNK1 both in the neural tube and during migration (Figures 10G–10H). The amount of departing cells is very high which as a result massively reduces the size of the neural tube on the electroporated side. These data show that coelectroporation of *h-ets-1* and *snail-2* is sufficient to induce EMT followed by massive dispersion of migratory NCCs from the intermediate part of the neural tube. To further characterize the nature of the specific cooperation between ets-1 and snail-2, we analysed the effect of *snail-2* electroporation alone in the neural tube at 48hpe. Interestingly, snail-2 is able to induce ectopic neural crest fate as shown by ectopic HNK1 staining (Figures 10M–10N, n = 6) but fails to provoke ectopic delamination or EMT and has no effect on N-cadherin expression or localisation (Figures 10I–10L, n = 4). Those results indicate that ectopic NCC fate in ets-1 and snail-2 coelectroporated embryos is due to snail-2 alone. However, the massive ectopic EMT that occurs in coelectroporated embryos is specific to ets-1 and snail-2 cooperation as it is never detected when either ets-1 or snail-2 are independently electroporated.

## Discussion

**Figure 11:**
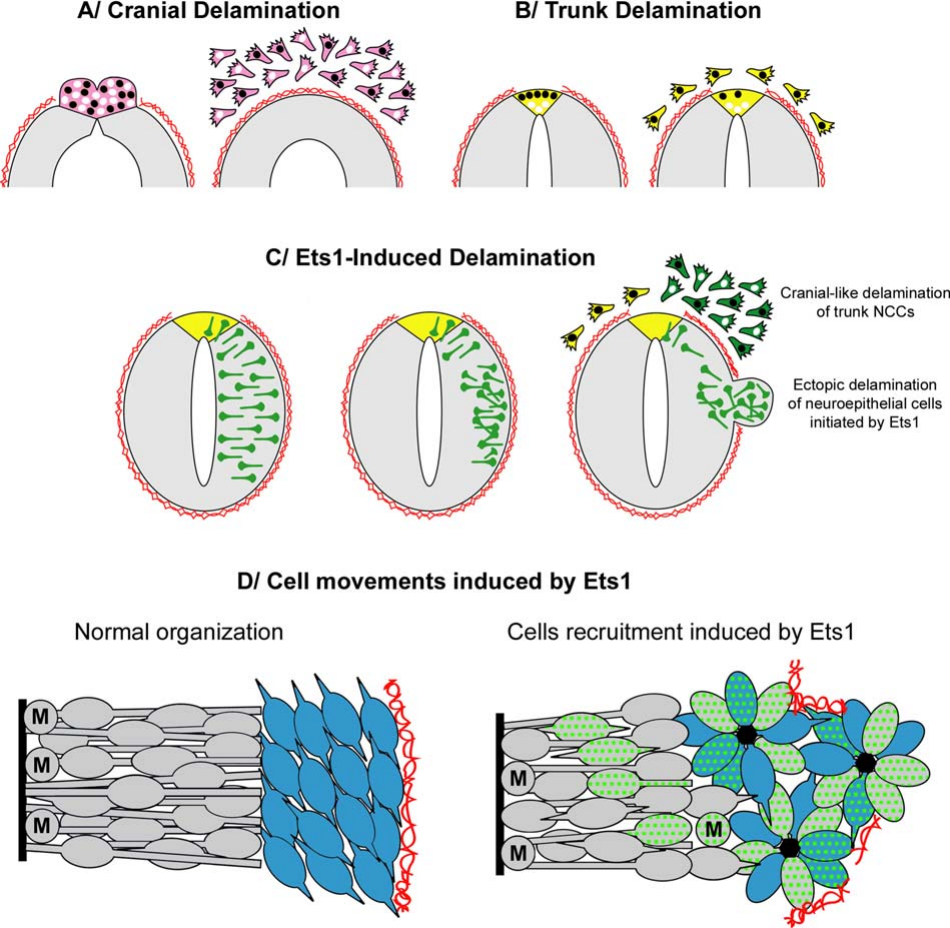
Ets-1 confers cranial features on neural crest delamination. (A) Normal delamination of cranial NCCs. Premigratory and migratory NCCs expressing *ets-1* are in purple. (B) Normal delamination of trunk NCCs. Premigratory and migratory NCCs are in yellow. (C) Consequences of *ets-1* electroporation in trunk neural tube at dorsal and at intermediate to ventral levels. *Ets-1* electroporated cells are coloured in green. (D) Cell movements induced by *ets-1* expression. Proliferating cells are in grey, non-proliferating cells are in blue. *Ets-1* electroporated cells are dotted in green. Cell-cell junctions involving N-cadherin are represented by black centers. Nuclei in S-phase are colored in black. Basal lamina is represented by twisted red line. Cranial NCCs express *ets-1* and massively delaminate independently of G1/S transition (A) whereas trunk NCCs do not express *ets-1* and delaminate progressively as a cell population subjected to successful G1/S transition (B). When *ets-1* expression is forced in the dorsal part of trunk neural tube, trunk NCCs delamination is greatly enhanced and cells emigrate as multilayered streams (C, green cells). Moreover, they lose their subjection to cell cycle progression indicating that *ets-1* converts trunk delamination into cranial-like emigration (C). Ectopic *ets-1* expression in ventral part of the neuroepithelium leads to massive cell movements without affecting cell proliferation or differentiation. Electroporated cells are accumulated close to the basal side of the neural tube and the basal lamina is degraded (C, D). These events are sufficient to initiate delamination. However, other factors such as *snail-2* are required to perform full delamination and promote EMT and cell migration. M, cell in mitosis.

Here, we more precisely describe the delamination of the cranial NCCs and find that these cells are first massively gathered at the dorsal part of the neural tube before the onset of migration and that their following delamination is characterized by emergence of great numbers of cranial NCCs in multilayered streams of cells (Figure 11A). Importantly, we show that these cells, in contrast to trunk, are not synchronized in S-phase (Figures 11A–11B). Therefore, the kinetic features of the cranial delamination are characterized by great number of cells delaminating at the same time and absence of S-phase subjection. We provide evidence that *ets-1*, which is specifically expressed by cranial NCCs, holds a pattern perfectly matching early phases of cranial delamination and plays a central role in this process. *Ets-1* is necessary for proper cranial NCCs delamination since inhibition of its activity in cephalic neural tube results in a great reduction or even prevention of cranial crest delamination. At trunk level, NCCs delaminate one after the other with the restriction they have successfully achieved their G1/S transition ([11]; Figure 11B). Strikingly, *ets-1* misexpression in the dorsal trunk is sufficient to convert the parcimonious outflow of isolated NCCs in S-phase into massive cranial-like delamination of unsynchronized cells (Figure 11C). We also show that ectopic *ets-1* electroporation in intermediate to ventral regions of trunk neural tube, a region normally unable to produce NCCs, leads to massive mobilization of neuroepithelial cells along the apico-basal axis of the neural tube, associated with local degradations of the basal lamina and initiation of ectopic delamination (Figures 11C–11D). These phenomenon occur without changing original identity of the cells or their proliferation rate. Electroporated cells do not undergo EMT and thus hold no migratory capabilities. Alone, *ets-1* is therefore able to perform cell sorting and to induce selected cells to disrupt the basal lamina. These events are sufficient to initiate but not complete the delamination process. In addition, we show that *ets-1* can cooperate with other genes to achieve full delamination since when coelectroporated with *snail-2* in the trunk neural tube, coelectroporated cells acquire migratory NCCs identity, massively leave the neural tube by an EMT process and migrate away.

**Figure 12:**
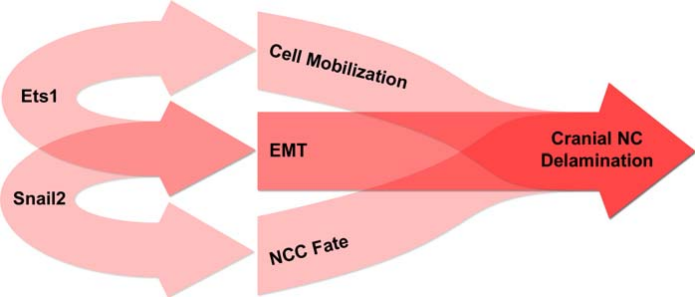
Ets-1 and Snail-2 cooperate to achieve the cranial NCC delamination.

Altogether, these results lead us to conclude that *ets-1* is necessary and sufficient to confer cranial features to NCCs delamination independently of neural crest induction and suggest that *ets-1* and *snail-2* cooperate to achieve the cranial NCC delamination (Figure 12)

### *Ets-1* Acts Independently of Neural Crest Cells Induction

Here, we show that electroporation of the dominant negative *c-ets-1 DBD* leads to dramatic reduction of cranial emigrating NCCs which do not delaminate. Conversely, when misexpressed in trunk dorsal region of the neuroepithelium, *h-ets-1* dramatically enhances trunk NCC delamination by anticipating their departure, increasing their number and extending duration of their exit. This raised the question of whether *ets-1* would play a role in NCCs induction with consequences on delamination or only regulates delamination.

NCC ontogeny proceeds in sequential steps including specification of NCC precursors territory, acquisition of premigratory NCC identity, delamination from the neural tube and migration in the periphery. Previous data have shown that some of these events are independent from each other. For instance, *foxd-3* ectopically expressed in intermediate to ventral neural tube induces NCCs markers but this induction is not followed by EMT [37], [38]. Also, blockade of delamination does not interfere with NCC specification [8], [10]. This seems also to be the case here as inhibition of delamination by *c-ets-1 DBD* happens without affecting NCC specification, *ap-2, snail-2, foxd-3 and sox-9* remaining expressed in premigratory NCCs, within the neural tube. This implies that cephalic premigratory NCCs require ETS-1 activity only to delaminate. However, the fact that *h-ets-1* misexpression enhances trunk NCCs delamination could be interpreted as an enlargement of the NCC territory at the expense of the intermediate region of the neural tube. Importantly, this capacity of *h-ets-1* to expand NCC flow is very restricted along dorsoventral axis of the neural tube. This suggests that *ets-1* alone cannot displace the ventral limit of the territory competent to produce NCCs and indicates that expression patterns of genes responsible for NCCs specification are not enlarged by *ets-1*. Therefore, in the dorsal trunk, one can suggest that *h-ets-1* increases the total amount of delaminating NCCs by recruiting at once all or a large part of the NCC precursors present in the dorsal neural tube. However, in this case, *ets-1* would be only able to prime but not to prolong the trunk delamination because of the rapid exhaustion of the premigratory NCCs population. Consequently, we alternatively suggest that *ets-1* continuously recruits neuroepithelial cells from intermediate regions of the neural tube and leads them to enter into the NCCs territory where they are specified. This is consistent with the observation that, two days after electroporation, when the dorsal region has lost its ability to produce NCCs, the electroporated cells leaving the dorsal neural tube are not NCCs anymore.

Overall, these findings show that *ets-1* is a major actor of the delamination process and it acts independently of NCCs specification.

### *Ets-1* Expression Abolishes Requirement of Successful G1/S Transition During Trunk NCCs Delamination

*Ets-1* electroporated trunk NCCs delaminate into massive streams independently of the S-phase of the cell cycle. This is in contrast to normal situation where trunk NCCs delaminate one by one and where it has been shown that only NCCs in S-phase are able to exit the neural tube [11]. This process is under the regulation the Bmp/Wnt pathway [10]. It could be argued that S-phase is the most favorable phase to delaminate as S-phase nuclei are located at the basal side of the neuroepithelium from which cells exit [11]. In agreement with this argument, since premigratory NCCs are not synchronized in S-phase prior delamination, the outflow of trunk NCCs is restrained as few cells are in the appropriate phase of the cell cycle. However, when the G1/S transition is blocked in vivo, no nucleus-free zone can be detected at the border of the neural tube [10], [11]. Moreover, cranial NCCs which naturally express *ets-1* delaminate at high rate without being in S-phase. Similarly, when they misexpressed *h-ets-1*, trunk NCCs delaminate massively, even when they are not in S-phase. Therefore, these results suggest that the position of cells within the neuroepithelium is not the sole explanation for S-phase requirement. Interestingly, it has been previously described that some promoters are only accessible for transcription factors during S-phase thanks to the loose chromatine organization during the DNA replication [39]. In trunk NCCs, promoters of some targets of the Bmp4/Wnt1 cascade might be only accessible during S-phase. This would explain the unique ability of these cells to leave the neural tube. When they misexpress *ets-1*, they are able to bypass the S-phase condition and delaminate.

Altogether, these data raise the question of the putative subjection to G1/S transition of the remaining cranial NCCs when ETS-1 activity is inhibited. There are numerous genes and mechanisms involved in the control of G1/S transition during trunk NCCs delamination including in particular Bmp/Wnt signaling pathway ([10], [30], for review see [7]). This regulation is strongly dependent of specific interactions occuring between trunk neural tube and somites which are lacking in cranial regions. In addition, expression patterns and identified roles of the members of Bmp and Wnt pathways are different from those known at trunk level [40]-[44]. Therefore, G1/S subjection of cranial NCCs when endogenous ETS-1 activity is inhibited seems unlikely.

### *Ets-1* is Responsible for the Particular Kinetics of Cranial Delamination

We show that *ets-1* is sufficient to initiate ectopic delamination process by recruiting massively neuroepithelial cells and by inducing a cranial-like departure of trunk NCCs. Conversely, inhibition of endogenous ETS-1 activity after *c-ets-1 DBD* misexpression abolishes the massive delamination of cranial NCCs. These results strongly indicate that *ets-1* is responsible for the particular kinetics of the cranial delamination. However, one could hypothesize that *c-ets-1 DBD* blocks other members of the ETS family in addition to ETS-1. Nevertheless, the inhibitory effect of *c-ets-1 DBD* on NCCs delamination is restricted to the cranial region where *ets-1* is the sole member of ETS family known to be expressed at the time of NCCs departure. Moreover, co-electroporation of *c-ets-1 DBD* and *h-ets-1* perfectly reverses the phenotype induced by *h-ets-1*. Then, we argue that the blockade of the cranial NCCs delamination, caused by the *c-ets-1 DBD* electroporation, is due to the lack of endogenous ETS-1 activity.

Our results indicate that when misexpressed in the neuroepithelium, *h-ets-1* induces ectopic delaminations of packed clusters of transfected cells barely mingling with non-electroporated cells. This process is characterized by nuclei accumulation on the basal side of the neural tube without increase of cell proliferation or loss of cell original identity. At the contrary, cells transfected with an inactive mutant form (*w375r*) are spaced out and randomly distributed within the neuroepithelium. Therefore, *ets-1* holds the ability to sort out cells from a population. It is also able to initiate their delamination since the massive recruitment of cells is associated with local degradation of the basal lamina. Our results suggest that *ets-1* might act by modifying the expression of cell surface adhesion molecules mediating cell-cell recognition and cluster formation. Indeed, we observe reorganization of the neuroepithelium with loss of pseudostratified layout of the neural tube, downregulation of *cadherin-6B* expression and perturbed distribution of N-cadherin. We also detecte bi-directional emigration of the transfected cells, both towards the lumen and the basal lamina, reminiscent of a defect of apicobasal polarity of the neural tube obtained by an increase or a decrease of cadherin expression [45]. However, *ets-1* alone does not allow cells to undergo EMT and does not bestow them with migratory capabilities. The ectopic bulges remain attached to the neural tube and delamination is not completed. Therefore, *ets-1* cannot summarize all the aspects of cranial delamination. Here, we have shown that *ets-1* and *snail-2* cooperate to induce ectopic EMT. Similarly, a cooperation between *ets-1* and an other member of the Snail family (*snail-1*) has been previously described in human squamous carcinoma cells [46]. All these results suggest that *ets-1* expressed in cranial NCCs might also synergize with *snail-2* to induce full delamination process (Figure 12). Mechanisms which support this cooperation remain to be elucidated.

Altogether, our results show that, at cranial level, delamination is the result of two separable cellular events: (i) a massive mobilization of premigratory NCCs orchestrated by *ets-1* that enables them to sort themselves out within the neuroepithelium and to acquire the ability to delaminate massively and (ii) a proper mesenchymalization controled by multiple genes.

## Materials and Methods

### Embryos

Fertilized eggs from Fasso strain chickens (brown eggs) were incubated at 38°C for appropriate times, then windowed and staged according to Hamburger and Hamilton [47].

### Plasmid constructs, in ovo electroporation, cell death and BrdU labeling

Full-length human *ets-1* cDNA (kindly provided by J. Ghysdael) was inserted downstream of adenovirus enhancer and RSV promoter in pAdRSV expression plasmid [48]. Integrity of the sequence was verified by restriction maps and sequencing. Level of expression and molecular weight of the encoded protein was checked by immunoblots performed on extracts of transiently transfected 293 cells. Nuclear localization of the protein was also asserted by immunochemistry in the same cells using a polyclonal anti-ETS-1 (gift of J. Ghysdael). An inactive mutant, *h-ets-1 w375r*, unable to bind DNA and to transactivate expression [49], [50] was generated by transforming tryptophan in position 375 of h-ETS-1 into arginine by PCR mutagenesis. A non-phosphorylable form of *ets-1* (*h-ets-1 t38a*) [51] in which threonine in position 38 is replaced by alanine was created by PCR mutagenesis. A dominant negative form, *c-ets-1 DBD*, was created by inserting the chick *ets-1* DNA-binding domain corresponding to amino acids 306 to 423 into pAdRSV by PCR amplification. The resulting protein binds target DNA but, lacking its transactivation domain, does not transactivate expression [52]. We checked efficiency of *c-ets-1 DBD* by testing its ability to inhibit ectopic delaminations induced by *h-ets-1*. After, coelectroporation of *c-ets-1DBD* and *h-ets-1* in the trunk of stage HH14 embryos, we did not find any ectopic delaminations at 48hpe (n = 5, data not shown). Plasmid driving full lenght chick *snail-2* expression was provided by J. Briscoe and M. Cheung. Embryos were electroporated between stages HH7 and HH10 for head and HH10+ and HH14 for trunk and collected as indicated. Plasmids encoding *h-ets-1*, *h-ets-1w375r* or *c-ets-1 DBD* were co-electroporated with a plasmid encoding enhanced GFP (pCAβ-EGFP; gift of J. Gilthorpe) at respectively 2 µg/µl and 1 µg/µl in 12% sucrose solution containing 0,1% Fast-Green (Sigma). Plasmid solution was mouth pipetted into the lumen of the neural tube with a stretched glass capillary, anteriorward from the level of approximately the third somite for head and last somite for trunk. Electrodes (CUY610 platinum-coated, NEPA Gene) were applied on vitelline membrane on each side of the tube at level of the injection. A square wave stimulator was used to deliver 4 pulses of 50 ms and 18V (head) or 35V (trunk) at a frequency of 2 Hz unilaterally. Embryos were allowed to develop to specified stages, harvested in phosphate buffered saline (PBS), monitored for GFP fluorescence and fixed in paraformaldehyde (PFA, 4% in PBS). After electroporation with *h-ets-1*, no important cell death was detected at 12hpe, 18hpe, 24hpe and 48hpe by TUNEL (Roche, n = 5), Nile blue staining (Sigma, n = 13) and DAPI staining (sigma, n = 15). For S-phase analysis, vitelline membrane was punctured and embryos received in ovo 25-100 µl of 0,2 mg/ml BrdU (Sigma) 1 hour before harvesting.

### Immunohistochemistry

Immunochemical detections of proteins were performed on cryosections of embryos fixed 1 hour at room temperature (RT) or overnight at 4°c in 4% PFA with following primary antibodies and dilutions: anti-BrdU 1∶100 (Becton Dickinson), anti-Cadherin-7 1∶200 (Gift of S. Nakagawa), anti-N-cadherin 1∶500 (Sigma), antifibronectin 1∶500 (Gift of K. Yamada), anti-GFP 1∶500 (Sigma), anti-Phospho-Histone-H3 1∶100 (Upstate Biotech.), anti-HNK-1 1∶25 (N. Desban and JLD), anti- β1 integrin 1∶100 (Gift of K. Yamada), anti-activated β1 integrin 1∶100 (TASC 9D11, Chemicon), anti-β3 Tubulin (Tuj1, Chemicon), anti-Laminin 1∶50 (Gift of H. Kleinman), anti-Lim1/2 (4F2), anti-Pax6, anti-Pax7 and anti-Tenascin (M1B4) 1∶100 (Developmental Study Hybridoma Bank). Following secondary antibodies were used at a 1∶100 dilution: anti-mouse Ig-Biot, anti-sheep IgG-Biot, anti-goat IgG-Biot (Sigma), anti-mouse IgG1-Texas Red, anti-mouse IgMBiot (Southern Biotechnology Associates), anti-mouse IgM-Alexa 488 (Molecular Probes). Briefly, slides were degelatinized, blocked in 2% FCS, incubated 2 hours at RT in primary antibody, 1 hour with secondary antibody and 1/2 hour with coupled-streptavidin 1∶500 (Molecular Probes and Southern Biotechnology Associates) if necessary. For BrdU detection, sections were incubated 1 hour in 5% 1 M trisodium citrate pH 6.7/95% formamide at 65°C. Blocking and antibodies incubations were carried out in 0.25% triton X100. DAPI (Sigma) and Phalloidin-TRITC (Sigma) used to stain nuclei and actin microfilaments respectively, were directly applied on cryosections for 15 minutes. In some cases, sections were stained by May-Grünwald Giemsa solutions (Merck) or assayed for TUNEL (Roche). Nikon Eclipse E800 microscope with Nikon DMX 1200 F camera was used to capture pictures.

### Whole mount in situ hybridization

Whole mount RNA in situ hybridization was performed using either non radioactive digoxigenin (DIG) probe for single labeling, or both DIG- and FITC-labeled RNA probes for double labeling with chick-specific probes *ap-2* (J. Richman), *cad-6B* and *cad-7* (M. Takeichi), *cyclinD-1* (J. Lahti), *ets-1* (B. Vandenbunder), *β3-integrin* [35], *foxd-3* (C. Erickson), *rhob* (Y. de Curtis), *snail-2* (A. Nieto), *sox-2* (P. Sharpe), *sox-9* (J. Briscoe), *sox-10* (P. Scotting) and human-specific *ets-1* (J. Ghysdael). Reaction was carried out essentially as described by Wilkinson 1992 [53] except that proteinase K steps were omitted. Dark staining was obtained using NBT/BCIP reagents (Boehringer Mannheim) whereas light blue staining was obtained using BCIP alone. Specimens were refixed using 4% PFA prior to storing or sectioning. For sectioning, embryos were infiltrated with 15% sucrose and embedded in 20% gelatin solution in PBS. Blocks were refixed 24 hours in 4% PFA/0.1% glutaraldehyde transversely sectioned on a vibratome (Leica) at 30 µm and further cleared in 60% glycerol/PBS. For whole mounts, images were collected on Nikon SMZ1500 and Leica MZFL III stereomicroscopes equipped with diascopic stand and Nikon DMX 1200 camera. For sections, Nikon Eclipse E800 microscope with Nikon DMX 1200 F camera was used.

### Measurements of cell proliferation

To establish the ratio of neuroepithelial cells in S-phase out of the total number of cells, DAPI and BrdU positive cells were counted on 5 µm cryosections in fields of at least 1600 µm2. In each case, at least 3 embryos and 5 non-adjacent sections per embryo were used for analysis. In electroporated embryos, cells were scored in the GFP area and in corresponding area on control side in order to compare electroporated with non-electroporated regions (h-ets-1: n(embryos) = 3, n(transfected cells) = 1036, n(control cells) = 1198); w375r: n(embryos) = 3, n(transfected cells) = 1173, n(control cells) = 1257). In normal embryos, cells were counted in regions corresponding to endogenous chick *ets-1* expression during (n = 9; n(mid cells) = 1479; n(sur cells) = 1904) and after delamination (n = 6; n(del cells) = 624; n(mid cells) = 659; n(sur cells) = 1037) of cranial NCCs. In these embryos, surrounding regions of the neural tube were used as reference.
